# P02.163. Acupuncture and donor egg *in vitro* fertilization cycles: a retrospective chart review comparing two acupuncture protocols

**DOI:** 10.1186/1472-6882-12-S1-P219

**Published:** 2012-06-12

**Authors:** L Hullender Rubin, M Opsahl, D Ackerman

**Affiliations:** 1 Oregon College of Oriental Medicine, Research Department, Portland, USA; 2 Northwest Center for Reproductive Medicine, Kirkland, USA

## Purpose

Research investigating the effect of acupuncture on *in vitro* fertilization (IVF) pregnancy outcomes has focused primarily on non-donor cycles. Most trials excluded donor IVF cycles in their design, as donor IVF live birth outcomes are higher than non-donor IVF. In this retrospective chart review, we compared the effect of two standardized acupuncture protocols on donor IVF live birth outcomes.

## Methods

Live births were compared between patients who elected acupuncture treatment before and after embryo transfer (ET) to those who did not. Acupuncture was performed at a private infertility clinic the same day as ET by one of seven licensed acupuncturists. One hundred thirty-four patients had an embryo transfer, of which 43 elected acupuncture. The acupuncture group “A” (Acu A) received the Craig protocol (Paulus protocol plus CV-6/Qihai before and KI-3/Taixi after ET) from 2005-2007 (N=23). From 2008-2009, the acupuncture group “B” (Acu B) received the Paulus protocol modified only with CV-6/Qihai added pre-ET (N=20). Live birth outcomes were analyzed using crude risk ratios.

## Results

There were 20 (87%) live births in the Acu A Group and 31 (66%) in the No Acu A group (RR=1.32, 95% CI 1.02 – 1.71, p=0.04). After removing KI-3/Taixi from the acupuncture protocol, there were 10 (50%) live births in the Acu B group and 28 (64%) in the No Acu B group (RR=1.37, 95% CI 0.76 – 2.47, p=0.29). When comparing Acu A to Acu B, there were significantly more live births in the Acu A group (RR=1.74, 95% CI 1.09 – 2.77, p=0.02).

## Conclusion

Donor IVF live births may be improved with the Craig acupuncture protocol. This finding should be taken cautiously as more rigorous research including randomization and a larger sample size is needed.

